# Immunogenicity of E2CD154 Subunit Vaccine Candidate against Classical Swine Fever in Piglets with Different Levels of Maternally Derived Antibodies

**DOI:** 10.3390/vaccines9010007

**Published:** 2020-12-24

**Authors:** Yusmel Sordo-Puga, Danny Pérez-Pérez, Carlos Montero-Espinosa, Aymé Oliva-Cárdenas, Iliana Sosa-Teste, Carlos A. Duarte, María Pilar Rodríguez-Moltó, Talía Sardina-González, Elaine Santana-Rodríguez, Milagros Vargas-Hernández, Yaneris Cabrera-Otaño, Julio A. Ancizar-Fragoso, Yohandy Fuentes-Rodríguez, Mario Pablo Estrada, Marisela Suárez-Pedroso

**Affiliations:** 1 Centro de Ingeniería Genética y Biotecnología, La Habana, 10600 Cubanacán, Cuba; yusmel.sordo@cigb.edu.cu (Y.S.-P.); danny.perez@cigb.edu.cu (D.P.-P.); carlos.montero@cigb.edu.cu (C.M.-E.); ayme.oliva@cigb.edu.cu (A.O.-C.); carlos.duarte@cigb.edu.cu (C.A.D.); pilar.rodriguez@cigb.edu.cu (M.P.R.-M.); talia.sardina@cigb.edu.cu (T.S.-G.); elaine.santana@cigb.edu.cu (E.S.-R.); milagros.vargas@cigb.edu.cu (M.V.-H.); yohandy.fuentes@cigb.edu.cu (Y.F.-R.); mario.pablo@cigb.edu.cu (M.P.E.); 2 Centro Nacional para la Producción de Animales de Laboratorio, La Habana, 10600 Cubanacán, Cuba; iliana.sosa@cenpalab.cu; 3 Instituto de Investigaciones Porcinas, 19200 Artemisa, Cuba; yaneriscabrera72@gmail.com (Y.C.-O.); jancizarjr@gmail.com (J.A.A.-F.)

**Keywords:** classical swine fever virus, E2CD154, maternally derived antibodies, subunit vaccine candidate

## Abstract

E2CD154 is a novel subunit vaccine candidate against classical swine fever virus (CSFV). It contains the E2 envelope protein from CSFV fused to the porcine CD154 molecule formulated in the oil adjuvant Montanide^TM^ ISA50 V2. Previous works evidenced the safety and immunogenicity of this candidate. Here, two other important parameters related to vaccine efficacy were assessed. First, the existence of high maternally derived antibody (MDA) titers in piglets born to sows vaccinated with E2CD154 was demonstrated. These MDA titers remained above 1:200 during the first seven weeks of life. To assess whether the titers interfere with active vaccination, 79 piglets from sows immunized with either E2CD154 or a modified live vaccine were vaccinated with E2CD154 following a 0–21-day biphasic schedule. Animals immunized at either 15, 21, or 33 days of age responded to vaccination by eliciting protective neutralizing antibody (NAb) titers higher than 1:600, with a geometric mean of 1:4335, one week after the booster. Those protective levels of NAb were sustained up to six months of age. No vaccination-related adverse effects were described. As a conclusion, E2CD154 is able to induce protective NAb in piglets with different MDA levels and at different days of age.

## 1. Introduction

Classical swine fever (CSF) is a highly contagious viral disease that affects domestic and wild pigs, and is considered to be one of the infectious diseases with the greatest economic impact in the pig industry worldwide [1,2,3]. It is a World Organization for Animal Health (OIE) notifiable disease, and enzootic in Eastern Europe, Southeast Asia, South America, Central America, and the Caribbean. CSF-derived losses in the pig industry contribute to the economic and social deterioration of developing nations [4,5]. The etiological agent of this disease is classical swine fever virus (CSFV), an enveloped RNA pestivirus from the *flaviviridae* family that is 40 to 60 nm in diameter with hexagonal symmetry [6].

Modified live vaccines (MLV) have been found to be effective in protecting pigs against CSFV and have been extensively used in endemic areas. However, frequent CSFV outbreaks have been described in many countries where MLV have been applied [7,8,9]. Therefore, one of the main objectives of the OIE-FAO Americas Program for CSF is the development of novel marker subunit vaccines against CSF, with some improved characteristics in relation to the currently available MLV.

E2CD154 is a novel subunit vaccine candidate against CSF. Its active principle is a recombinant chimeric antigen formed by the extracellular region of the E2 glycoprotein fused to the swine CD154 molecule (E2CD154). This vaccine candidate has proven to be safe and immunogenic in weaning piglets. In addition to a strong neutralizing antibody (NAb) response, E2CD154 induces interferon gamma-secreting T cells as early as seven days after vaccination. Unlike previously described subunit immunogens, E2CD154 confers early-onset protection against a challenge with a virulent CSFV strain [10]. The absence of viral RNA in the target organs, nasal/rectal exudates, and serum of the challenged pigs evidences the capacity of this vaccine candidate to inhibit viral replication and therefore to prevent horizontal transmission [10]. E2CD154 can also efficiently prevent vertical transmission in pregnant sows after viral challenge, a goal that was elusive for previous E2-based subunit vaccine candidates [11].

Since the aim of this vaccine study was to introduce E2CD154 into areas that were already vaccinated with MLV, other relevant aspects must be assessed during the clinical evaluation of this vaccine candidate. One of these aspects is the transmission of protective levels of NAb from the colostrum of vaccinated sows to their offspring during the first days of life. Another important issue is to demonstrate that maternally derived antibodies (MDA) do not interfere with the immune response of piglets at the moment of vaccination, which is described as an advantage of subunit vaccine candidates as compared to MLV.

The aim of this work was to study the levels of MDA in piglets born to E2CD154-immunized sows, and to assess the efficacy of E2CD154 in piglets of different ages and with diverse MDA levels born to sows immunized with MLV with or without E2CD154 vaccination.

## 2. Materials and Methods

### 2.1. E2CD154 Vaccine Candidate

Briefly, the active ingredient of E2CD154 is a chimeric protein formed by the fusion of the extracellular region of the E2 glycoprotein in the Margarita CSFV strain [1] and the extracellular segment of the swine CD154 molecule. A lentivirus-based gene delivery system was used to generate a stable recombinant HEK 293 cell line (ATCC CRL1573) for the expression of the E2-CSFV antigen fused to the porcine CD154 molecule, as previously described [10]. The E2CD154 protein was formulated in Montanide^TM^ ISA50 V2 (SEPPIC, France) using a 60/40 proportion of aqueous/oil phase. The water-in-oil emulsion was produced with an SD-41 homogenizer (IKA, Germany) under good manufacturing practice (GMP) conditions. The concentration of E2CD154 in the final emulsion was 25 μg/mL.

### 2.2. Experimental Animals

Some of the animals used in this assay were kept in the areas of the National Center for the Production of Laboratory Animals (CENPALAB, La Habana, Cuba) under controlled conditions. Others were kept in the Institute for Porcine Research (IIP, Artemisa, Cuba) under swine production conditions following the established procedures [12]. Research protocols were approved by the Committee for the Care and Use of Laboratory Animals (CICUAL) at CENPALAB (reference number 24/15).

### 2.3. Immunization Schedule

E2CD154 (50 μg of E2CD154 in 2 mL of Montanide^TM^ ISA50 V2) was administered via a deep intramuscular route in the neck. A two-dose immunization scheme was followed. The first immunization was performed at day 0 on the right side of the neck and the second at day 21 on the left side, using 18 G × 1 inch needles in compliance with good veterinary clinical practices.

### 2.4. Time Course of MDA

Six offspring from one sow immunized only with E2CD154 during the first trimester of pregnancy were studied in controlled conditions in an experimental station. Animals were sampled at 12, 26, 38, 45, 52, 59, 66, and 73 days after birth to evaluate the persistence of MDA. The NAb titer of the dam at farrowing was also measured.

Twenty piglets from two litters of sows immunized with MLV (Vacuna cólera porcino, LABIOFAM, Cuba) and re-vaccinated with E2CD154 during the first trimester of pregnancy were used to evaluate the time course of MDA in swine production conditions. Animals were sampled at 12, 26, 38, 45, and 52 days after birth to evaluate the persistence of MDA. The NAb titer of the sows at farrowing was also measured.

### 2.5. Interference of MDA with Vaccination

Twenty piglets from sows immunized exclusively with MLV (litters 125, 123, and 126) were vaccinated at weaning (33 days after birth) with E2CD154. Two experimental groups were formed from six other litters from sows immunized with MLV and revaccinated with E2CD154 in the first trimester of pregnancy, as described above. Three of those litters (114, 92, and 91, with a total of 29 piglets) received the first dose between day 17 and day 18 after birth, while the remaining three litters (112, 113, and 120, with a total of 30 piglets) received the first dose between day 19 and day 21 after birth. More detailed information about the vaccination schedules can be consulted in the Supplementary Materials. In all cases, NAb titers were monitored before vaccination (week 0) and in weeks 3, 4, 6, 16, and 25 after the first dose of the vaccine candidate. This experiment was carried out in field conditions.

### 2.6. Identification of the Animals

Animals were tagged in the paddocks with visible identification (notches) that identified the litters under study.

### 2.7. Neutralizing Antibody Detection

Serum samples were screened for the ability to neutralize the cell culture adapted Margarita CSFV strain (National Center for Animal and Plant Health, Mayabeque, Cuba) using NPLA [13], as described in the manual from the World Organization for Animal Health [5]. After inactivating the serum, dilute the serum in two-fold steps with a cell growth medium and an equal volume of a neutralizing test viral solution containing about 100 TCID/_50_ in 50 μL was added to each diluted serum and incubated at 37 °C for 60 min. A quantity of 50 µL of PK15 cells suspended at 8 × 104 cel/mL were added and incubated at 37 °C with 5% CO2 for 72 h and observed. The virus dilution, covering a range of 4 logarithms, was added to the neutralization plates and subjected to reverse titration. Back titration, which acts as an internal quality control, confirmed that the virus was used in a concentration between 30 and 300 DICT50/50 μL. Virus neutralization was detected by incubation with the anti E2 Mab CBSSE2.3 (CIGB-SS, Cuba) conjugated to horseradish peroxidase and incubated with 3-amino-9-ethyl carbazole (AEC) and hydrogen peroxide substrate. The presence of viral replication was determined by visual inspection with an optical microscope. The last serum dilution without any signal of virus replication was considered the neutralizing titer. Titers were expressed as the reciprocal of the higher dilution of serum that neutralized 100 TCID50 of Margarita strain in 50% of culture replicates. The results were expressed as the geometric mean (GM) of the NAb titers plus the confidence intervals.

### 2.8. Clinical Observation

Vaccinated animals were carefully evaluated daily for clinical signs, inappetence, prostration, inflammatory reactions at the inoculation site, appreciable changes in respiratory rate, and other alterations that may or may not have been related to the vaccination.

### 2.9. Statistical Analysis

Lineal regression equations for the GM of MDA titers versus time were calculated. The normal distribution of the data was assessed with the Kolmogorov–Smirnov and D’Agostino–Pearson tests. The Kruskal–Wallis test was used to compare antibody titers among the six groups of animals and Dunn’s multiple comparison test was used to look for individual differences among groups. The Mann–Whitney test was applied to compare antibody titers between two groups. The statistical package GraphPad Prism 6 was used for the entire analysis (Prism 6 for Windows, Version 6.01, GraphPad Software, Inc., La Jolla, USA). Statistical significance was considered when *p* < 0.05.

## 3. Results

### 3.1. Colostral Antibody Titers in Piglets from E2CD154-Vaccinated Sows

**Figure 1:**
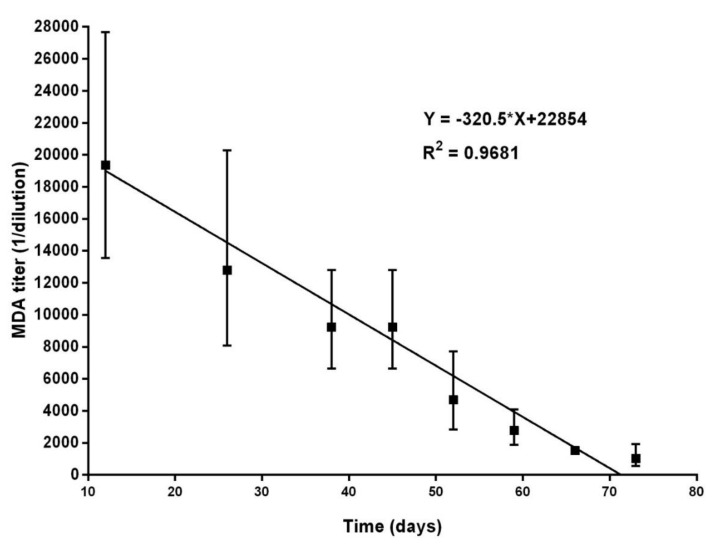
Serum NAb titers (MDA) in the offspring of one sow immunized with E2CD154. The sow received two injections of E2CD154 during the first trimester of pregnancy. Serum was collected on different days from six piglets and NAb titers were measured by NPLA. The GM and the confidence intervals of MDA titers are represented. The animals were kept in controlled conditions in an experimental station during the study.

First, the time course of MDA in the serum of the offspring from one sow immunized only with E2CD154 under controlled conditions was monitored (Figure 1). The NAb titer of the dam at farrowing was higher than 1:25,600. MDA titers with a GM higher than 1:19,367 were detected in the second week of life in piglets from this sow. The GM of MDA titers gradually decreased until 73 days of age (10th week of life), when MDA titers with a GM of 1:1027 were still measured.

**Figure 2:**
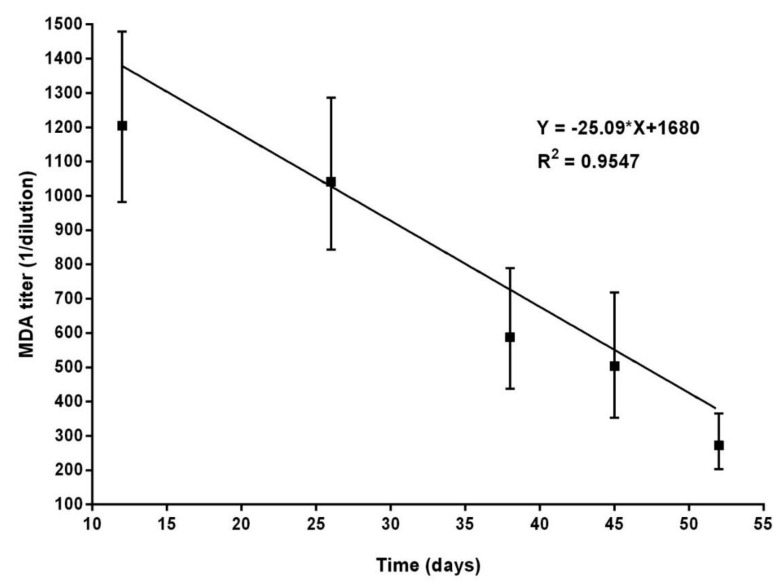
Serum NAb titers (MDA) in the offspring of two sows immunized with MLV and re-immunized with E2CD154. Sows received two injections of E2CD154 during the first trimester of pregnancy. Serum was collected on different days from twenty piglets and the NAb titers were measured by NPLA. The GM and the confidence intervals of MDA titers are represented. The study was conducted in swine under production conditions.

On the other hand, the group of piglets born from two sows immunized with MLV and re-vaccinated during pregnancy with E2CD154 under field conditions exhibited GM MDA titers of 1:1204 2 weeks after birth (Figure 2). The serum NAb titers of their corresponding sows at farrowing were 1:3200 and 1:6400. The MDA titers also decreased over time, but a GM of 1:272 was still found at 53 days of age (8th week of life).

### 3.2. Immunogenicity of E2CD154 in Piglets

#### 3.2.1. Immunogenicity of E2CD154 in Piglets with Low MDA Titers

**Figure 3:**
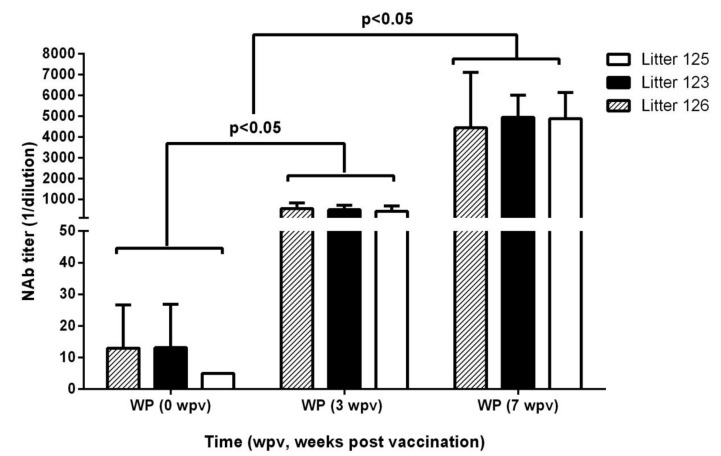
Serum NAb titers in piglets with low levels of MDA at the time of vaccination and immunized with two injections of E2CD154. First dose at weaning (33 days of age). Bars represent the GM and confidence intervals of the NAb titers, where wpv = weeks post vaccination and WP = weaning piglets. Kruskal–Wallis test was applied to compare NAb titers among the six groups and Dunn’s multiple comparison test was used to look for individual differences between groups.

The offspring of sows immunized only with the C-strain MLV, who received the vaccine candidate at weaning (five weeks of age, GM of titers 1:7.14), had low MDA titers when vaccinated and developed a strong NAb response, with GM values of 1:429 3 weeks after the first dose of E2CD154. A strong anamnestic response was observed after the booster, with the GM of the titer at 1:4335 by 7 weeks after the first dose of the vaccine (Figure 3). The individual NAb titers from all of the animals can be consulted in the Supplementary Materials.

#### 3.2.2. Immunogenicity of E2CD154 in Piglets with High MDA Titers

Next, the immunogenicity of E2CD154 in piglets born to sows additionally vaccinated with E2CD154 during pregnancy was explored. Those animals exhibited high MDA titers at the moment of vaccination.

**Figure 4:**
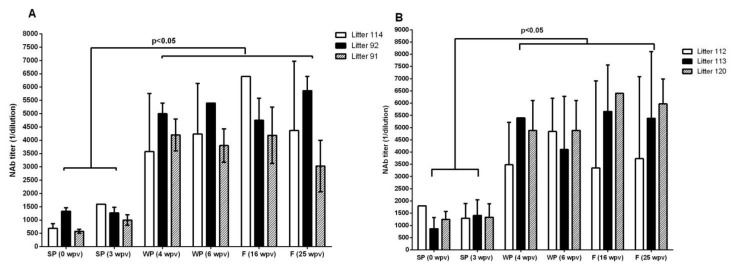
Serum NAb titers in piglets with high levels of MDA and immunized with two injections of E2CD154. (**A**) First dose at 15 days of age and (**B**) first dose at 21 days of age. Bars represent the GM and confidence intervals of the NAb titers. Wpv = weeks post vaccination, SP = suckling piglets, WP = weaning piglets, and F = fattening pigs. Kruskal–Wallis test was applied to compare NAb titers among the six groups and Dunn’s multiple comparison test was used to look for individual differences between groups.

The first group of piglets, vaccinated at 17–18 days of age, showed MDA titers with a GM of 1:780 at the moment of vaccination (Figure 4A). Those titers increased to 1:4017 after the booster (21 days post-prime vaccination) and remained above 1:3000 until the time of euthanasia (six months of age). Very similar results were observed in the animals immunized with E2CD154 at 19–21 days of age. These piglets, which had higher GM MDA titers (1:1262) when vaccinated, were able to respond to active vaccination by significantly enhancing their humoral immune response (Figure 4B). NAb titers reached GM values of 1:4496 after the booster and remained high until the time of euthanasia (six months of age).

Significant statistical differences were documented in both groups of animals one week after the booster (*p* < 0.05) compared to 0 and 3 weeks post vaccination (wpv), when the piglets had received only the initial dose of the vaccine. No significant statistical differences in the GM of the NAb titers between the two groups were documented before vaccination or at 42–43 (*p* = 0.7377), 113 (*p* = 0.4141), and 180 (*p* = 0.097) dpv. The individual NAb titers of all of the animals can be consulted in the Supplementary Materials.

## 4. Discussion

The passage of immunoglobulins to the fetus is limited in pigs due to their epitheliochorial type of placenta. As a consequence, neonates depend completely on maternally derived immune factors acquired during lactation for protection against pathogens [14]. High MDA titers (≥1:256) are required to protect pigs against a CSFV challenge during the early period of life [15]. Knowledge of the level and duration of maternal antibodies in the offspring of vaccinated sows is fundamental to determine whether there is a window of time before vaccination during which animals may be susceptible to CSFV infection. This knowledge is also fundamental to establish optimal timing for vaccination and to evaluate whether the presence of MDA interferes with vaccination.

The results presented in this paper demonstrate that E2CD154 is capable of inducing protective NAb titers in pregnant sows and that these translate to high titers of MDA in offspring. All piglets born from E2CD154-immunized sows exhibited MDA titers well above the protective threshold. The correlation between NAb titers in pregnant sows and MDA in suckling piglets was also reported in prior studies [16].

In the first experiment, conducted in an experimental setting with a controlled environment and ideal feeding conditions, very high MDA titers were found in the offspring of one sow immunized only with E2CD154. The level of MDA remained above the protective threshold until 73 days of age. More modest MDA titers were documented in a second experiment conducted on pig farms where the animals are in permanent contact with environmental and management factors that could affect their health. However, even with these differences, protective MDA titers were documented up to 53 days of age in the two litters of piglets studied.

The MDA titers found in piglets born to E2CD154-immunized sows were higher than those described elsewhere in the offspring of sows vaccinated with an E2 subunit marker vaccine candidate (from 1:32 to 1:512) [17]. These results coincide with those found by Vega et al., who showed that a subunit vaccine candidate based on the E2 glycoprotein was able to maintain high levels of MDA in offspring until the 8th week of life. Those authors also suggested that the duration of maternal immunity in animals vaccinated on farms may range from 6 to 9 weeks of age [18]. The same conclusion was derived from another study, where MDA titers were observed in offspring until 13 weeks of life [19].

Although a limited number of litters were included in this study, the data suggest that immunizing pregnant sows with E2CD154 guarantees the transfer of passive immunity to offspring during the first days of life until the time of standard vaccination practices. These results should be corroborated in further studies to fully demonstrate that E2CD154 vaccination provides a complete cycle of protection from pregnant sows to their offspring.

In the second part of this study, the immunogenicity of E2CD154 in piglets with different levels of MDA titers was assessed.

The negative influence of MDA in piglets on the immunogenicity of MLV is widely documented [20,21,22]. It was previously documented that MDA titers above 1:32 significantly affect the induction of protective immunity by a CSF MLV, leading to vaccine candidate failure [15,23,24]. The interference of MDA on MLV is due to the replicative nature of these immunogens. This can be problematic in endemic areas, where pigs are routinely vaccinated and CSFV circulation on farms is generally high.

The results reported by other authors suggest that the immunogenicity of subunit vaccine candidates containing the E2 protein is not affected by MDA titers at the moment of vaccination [25,26]. Immunization of two-week-old piglets with a subunit vaccine candidate induced protective NAb that reduced viral transmission at three and six months after vaccination. However, the NAb titers in this study were lower in piglets with MDA at the moment of vaccination, which suggests that protection could be less effective in those animals [17].

The robust immunogenicity of E2CD154 in animals with MDA was demonstrated here for the first time. The vaccine candidate was applied 2 to 3 weeks after birth, or at weaning, and high levels of protective NAb were elicited in all vaccinated piglets, independent of their MDA titers at the moment of vaccination. The high NAb titers observed after the two-dose vaccination of E2CD154 in piglets were similar to those described in animals immunized with the E2His protein [27]. However, those authors immunized weaning piglets at a much older age (56 days of life) and the experiment was developed in a controlled setting, whereas ours was conducted on a pig farm.

Importantly, it was demonstrated that the time to initiate vaccination with E2CD154 can be very flexible, as protective NAb responses were elicited in piglets vaccinated at 2–3 weeks of age or at weaning (around 5 weeks after birth). The feasibility of immunizing piglets of different days of age, and disregarding their levels of MDA, allows for the implementation of different vaccination schedules according to the practical needs of each farm. Vaccinating piglets around 15 days of age can be a recommendation derived from this study because it can confer a CSF protective status to the animals at the point when they may be transferred at weaning to other farms.

These results are therefore relevant from a practical point of view because they evidence the absence of a window of time with a lack of immunological protection in herds immunized with E2CD154. Suckling piglets will always maintain protective MDA (above 1:200) from their first lactation to the moment of vaccination. Another relevant aspect is that our experiments were conducted on pig farms, where animals are exposed to various conditions common in low-resource nations such as Cuba that can affect successful vaccination.

In summary, the immunogenicity demonstrated by this subunit vaccine candidate in piglets and the lack of interference from MDA in the vaccine candidate performance support the introduction of E2CD154 in endemic areas where conventional MLV are routinely applied.

## 5. Conclusions

Sows immunized with E2CD154 marker vaccine are able to transfer protective levels of maternally derived neutralizing antibodies to their offspring. Vaccination of those piglets with E2CD154 elicits high titers of neutralizing antibodies, notwithstanding the pre-existing levels of maternally derived antibodies.

## Supplementary Materials

The following are available online at https://www.mdpi.com/2076-393X/9/1/7/s1: Table S1: Piglets were vaccinated at 15 days of age. The sows were immunized with MLV and re-vaccinated with E2CD154 during the first third of pregnancy, Table S2: Piglets vaccinated at day 21 of age, the sows were inmunized with MLV and re-vaccinated with E2CD154 during the first third of pregnancy, Table S3: Piglets vaccinated at day 33 of age, the sows were inmunized only with MLV during the first third of pregnancy.
