# Supplementary material for: Immunogenicity of E2CD154 Subunit Vaccine Candidate against Classical Swine Fever in Piglets with Different Levels of Maternally Derived Antibodies
Source: Vaccines (Basel). 2020 Dec 24;9(1):7. doi: 10.3390/vaccines9010007 (PMC7823626; doi:10.3390/vaccines9010007)
Supplement: Supplementary file 1 [file vaccines-09-00007-s001.pdf]

Table 1: Piglets were vaccinated at 15 days of age. The sows were immunized with MLV and re-vaccinated with E2CD154 during the first third of pregnancy

Identification		0 WPV Vaccination: First dose				3 WPV Vaccination: Second dose				4 WPV			
sows	Piglets	Date	Age	Sample Code	NAb Titers	Date	Age	Sample Code	NAb Titers	Date	Age	Sample Code	NAb Titers
114	2	02/02/2016	18 days, suckling piglets	F7-11.1.02		23/02/2016	39 days, weaning piglets	F7-11.2.02	1600	08/03/2016	54 days, weaning piglets	F7-11.3.02	5400
	13			F7-11.1.13	800			F7-11.2.13	1600			F7-11.3.13	5400
	14			F7-11.1.14	800			F7-11.2.14	1600			F7-11.3.14	5400
	15			F7-11.1.15	800			F7-11.2.15	1600			F7-11.3.15	1800
	16			F7-11.1.16	400			F7-11.2.16	1600			F7-11.3.16	1800
	17			F7-11.1.17	800			F7-11.2.17	1600			F7-11.3.17	5400
	18			F7-11.1.18	800			F7-11.2.18				F7-11.3.18	
	19			F7-11.1.19	800			F7-11.2.19	1600			F7-11.3.19	1800
	20			F7-11.1.20	800			F7-11.2.20	1600			F7-11.3.20	5400
	21			F7-11.1.21	400			F7-11.2.21	1600			F7-11.3.21	
92	45	16/02/2016	17 days, suckling piglets	F7-12.1.53	1600	08/03/2016	38 days, weaning piglets	F7-12-2-45	1800	22/03/2016	52 days, weaning piglets	F7-12-3-45	5400
	46			F7-12.1.49	800			F7-12-2-46	1800			F7-12-3-46	5400
	47			F7-12.1.45	1600			F7-12-2-47	600			F7-12-3-47	5400
	48			F7-12.1.46	800			F7-12-2-48	1800			F7-12-3-48	5400
	49			F7-12.1.54	1600			F7-12-2-49	1800			F7-12-3-49	1800
	50			F7-12.1.51	1600			F7-12-2-50	600			F7-12-3-50	5400
	51			F7-12.1.47	1600			F7-12-2-51	600			F7-12-3-51	5400
	53			F7-12.1.50	1600			F7-12-2-53	600			F7-12-3-53	5400
	54			F7-12.1.48	800			F7-12-2-54	1800			F7-12-3-54	5400
91	87	01/03/2016	18 days, suckling piglets	F7-13.1.87	400	22/03/2016	39 days, weaning piglets	F7-13.2.87	600	05/04/2016	54 days, weaning piglets	F7-13.3.87	5400
	88			F7-13.1.88	800			F7-13.2.88	600			F7-13.3.88	5400
	89			F7-13.1.89	400			F7-13.2.89	1800			F7-13.3.89	1800
	90			F7-13.1.90	400			F7-13.2.90	1800			F7-13.3.90	5400
	92			F7-13.1.92	400			F7-13.2,92	600			F7-13.3,92	5400
	93			F7-13.1.93	800			F7-13.2.93	600			F7-13.3.93	5400
	94			F7-13.1.94	800			F7-13.2.94	600			F7-13.3.94	1800
	95			F7-13.1.95	800			F7-13.2.95	1800			F7-13.3.95	5400
	96			F7-13.1.96	400			F7-13.2.96	600			F7-13.3.96	1800

Identification		6 WPV				16 WPV				25 WPV			
sows	Piglets	Date	Age	Sample Code	NAb Titers	Date	Age	Sample Code	NAb Titers	Date	Age	Sample Code	NAb Titers
114	2	22/3/2016	69 days, weaning piglets	F7-11.4.02	5400	24/5/2016	129 days, fattening pigs	F7-11.5.02	6400	19/07/2016	189 days, fattening pigs	F7-11.6.02	2400
	13			F7-11.4.13	5400			F7-11.5.13				F7-11.6.13	
	14			F7-11.4.14	5400			F7-11.5.14	6400			F7-11.6.14	1600
	15			F7-11.4.15	5400			F7-11.5.15	6400			F7-11.6.15	6400
	16			F7-11.4.16	5400			F7-11.5.16	6400			F7-11.6.16	6400
	17			F7-11.4.17	1800			F7-11.5.17	6400			F7-11.6.17	6400
	18			F7-11.4.18				F7-11.5.18				F7-11.6.18	
	19			F7-11.4.19	5400			F7-11.5.19	6400			F7-11.6.19	3200
	20			F7-11.4.20	5400			F7-11.5.20	6400			F7-11.6.20	6400
	21			F7-11.4.21	1800			F7-11.5.21	6400			F7-11.6.21	6400
92	45	12/04/2016	73 days, weaning piglets	F7-12-4-45	5400	14/06/2016	135 days, fattening pigs	F7-12-5-45	6400	16/08/2016	197 days, fattening pigs	F7-12-6-45	6400
	46			F7-12-4-46				F7-12-5-46				F7-12-6-46	
	47			F7-12-4-47	5400			F7-12-5-47	6400			F7-12-6-47	6400
	48			F7-12-4-48	5400			F7-12-5-48	6400			F7-12-6-48	6400
	49			F7-12-4-49	5400			F7-12-5-49	6400			F7-12-6-49	6400
	50			F7-12-4-50	5400			F7-12-5-50	6400			F7-12-6-50	6400
	51			F7-12-4-51	5400			F7-12-5-51	1200			F7-12-6-51	3200
	53			F7-12-4-53	5400			F7-12-5-53	3200			F7-12-6-53	
	54			F7-12-4-54	5400			F7-12-5-54	1600			F7-12-6-54	
91	87	19/04/2016	68 days, weaning piglets	F7-13.4.87	5400	21/06/2016	130 days, fattening pigs	F7-13.5.87	6400	23/08/2016	191 days, fattening pigs	F7-13.6.87	6400
	88			F7-13.4.88	5400			F7-13.5.88	6400			F7-13.6.88	3200
	89			F7-13.4.89	1800			F7-13.5.89	6400			F7-13.6.89	3200
	90			F7-13.4.90	1800			F7-13.5.90	1600			F7-13.6.90	800
	92			F7-13.4,92	5400			F7-13.5,92	300			F7-13.6,92	600
	93			F7-13.4.93	5400			F7-13.5.93	1800			F7-13.6.93	600
	94			F7-13.4.94	1800			F7-13.5.94	6400			F7-13.6.94	6400
	95			F7-13.4.95	5400			F7-13.5.95				F7-13.6.95	
	96			F7-13.4.96	1800			F7-13.5.96				F7-13.6.96	

Table 2: Piglets vaccinated at day 21 of age, the sows were inmunized with MLV and re-vaccinated with E2CD154 during the first third of pregnancy

Identification		0 WPV Vaccination: First dose				3 WPV Vaccination: Second dose				4 WPV			
Sows	Piglets	Date	Age	Sample Code	NAb Titers	Date	Age	Sample Code	NAb Titers	Date	Age	Sample Code	NAb Titers
112	77	23/02/2016	19 days, suckling piglets	F7-21.1.77	1800	15/03/2016	40 days, weaning piglets	F7-21.2.77	600	29/03/2016	54 days, weaning piglets	F7-21.3.77	1800
	78			F7-21.1.78	1800			F7-21.2.78	600			F7-21.3.78	5400
	79			F7-21.1.79	1800			F7-21.2.79	1800			F7-21.3.79	1800
	80			F7-21.1.80	1800			F7-21.2.80	1800			F7-21.3.80	5400
	81			F7-21.1.81	1800			F7-21.2.81	1800			F7-21.3.81	1800
	82			F7-21.1.82	1800			F7-21.2.82	1800			F7-21.3.82	5400
	83			F7-21.1.83	1800			F7-21.2.83	600			F7-21.3.83	1800
	84			F7-21.1.84	1800			F7-21.2.84	1800			F7-21.3.84	5400
	85			F7-21.1.85	1800			F7-21.2.85	1800			F7-21.3.85	5400
	86			F7-21.1.86	1800			F7-21.2.86	1800			F7-21.3.86	5400
113	66	23/02/2016	19 days, suckling piglets	F7-22.1.66	600	15/03/2016	40 days, weaning piglets	F7-22.2.66	1800	29/03/2016	54 days, weaning piglets	F7-22.3.66	5400
	67			F7-22.1.67	600			F7-22.2.67	1800			F7-22.3.67	5400
	68			F7-22.1.68	600			F7-22.2.68	600			F7-22.3.68	5400
	70			F7-22.1.70	600			F7-22.2.70	1800			F7-22.3.70	5400
	71			F7-22.1.71	600			F7-22.2.71	600			F7-22.3.71	5400
	72			F7-22.1.72	1800			F7-22.2.72	1800			F7-22.3.72	5400
	73			F7-22.1.73	1800			F7-22.2.73	1800			F7-22.3.73	5400
	74			F7-22.1.74	600			F7-22.2.74	1800			F7-22.3.74	5400
	75			F7-22.1.75	1800			F7-22.1.75	1800			F7-22.3.75	5400
120	55	23/02/2016	21 days, suckling piglets	(F7-23.1.55)	1600	15/03/2016	42 days, weaning piglets	F7-23.2.55	1800	29/03/2016	49 days, weaning piglets	F7-23.3.55	5400
	56			(F7-23.1.56)	800			F7-23.2.56	1800			F7-23.3.56	5400
	57			(F7-23.1.57)	1600			F7-23.2.57	1800			F7-23.3.57	5400
	58			(F7-23.1.58)	1600			F7-23.2.58	1800			F7-23.3.58	5400
	59			(F7-23.1.59)	1600			F7-23.2.59	1800			F7-23.3.59	5400
	60			(F7-23.1.60)	800			F7-23.2.60	1800			F7-23.3.60	5400
	61			(F7-23.1.61)	1600			F7-23.2.61	1800			F7-23.3.61	5400
	62			(F7-23.1.62)	1600			F7-23.2.62	600			F7-23.3.62	5400
	63			(F7-23.1.63)	800			F7-23.2.63	1800			F7-23.3.63	5400
	64			F7-23.1.64)	800			F7-23.2.64	600			F7-23.3.64	5400
	65			(F7-23.1.65)	1600			F7-23.2.65	600			F7-23.3.65	1800

Identification		6 WPV				16 WPV				25 WPV			
Sows	Piglets	Date	Age	Sample Code	NAb Titers	Date	Age	Sample Code	NAb Titers	Date	Age	Sample Code	NAb Titers
112	77	12/04/2016	68 days, weaning piglets	F7-21.4.77	5400	14/06/2016	129 days, Fattening pigs	F7-21.5.77	6400	16/08/2016	191 days, Fattening pigs	F7-21.6.77	6400
	78			F7-21.4.78	5400			F7-21.5.78	6400			F7-21.6.78	6400
	79			F7-21.4.79	5400			F7-21.5.79	600			F7-21.6.79	1600
	80			F7-21.4.80	5400			F7-21.5.80	6400			F7-21.6.80	6400
	81			F7-21.4.81	1800			F7-21.5.81	3200			F7-21.6.81	1600
	82			F7-21.4.82	5400			F7-21.5.82	3200			F7-21.6.82	6400
	83			F7-21.4.83	5400			F7-21.5.83	6400			F7-21.6.83	6400
	84			F7-21.4.84	5400			F7-21.5.84	800			F7-21.6.84	800
	85			F7-21.4.85	5400			F7-21.5.85	6400			F7-21.6.85	6400
	86			F7-21.4.86	5400			F7-21.5.86				F7-21.6.86	
113	66	12/04/2016	68 days, weaning piglets	F7-22.4.66	5400	14/06/2016	129 days, Fattening pigs	F7-22.5.66	6400	16/08/2016	191 days, Fattening pigs	F7-22.6.66	6400
	67			F7-22.4.67	5400			F7-22.5.67	2400			F7-22.6.67	1600
	68			F7-22.4.68	5400			F7-22.5.68	6400			F7-22.6.68	6400
	70			F7-22.4.70	5400			F7-22.5.70	6400			F7-22.6.70	6400
	71			F7-22.4.71	5400			F7-22.5.71	6400			F7-22.6.71	6400
	72			F7-22.4.72	5400			F7-22.5.72	6400			F7-22.6.72	6400
	73			F7-22.4.73	1800			F7-22.5.73	6400			F7-22.6.73	6400
	74			F7-22.4.74	1800			F7-22.5.74	6400			F7-22.6.74	6400
	75			F7-22.4.75				F7-22.5.75				F7-22.6.75	
120	55	12/04/2016	70 days, weaning piglets	F7-23.4.55	5400	14/06/2016	131 days, fattening pigs	F7-23.5.55	6400	16/08/2016	193 days, fattening pigs	F7-23.6.55	6400
	56			F7-23.4.56	5400			F7-23.5.56	6400			F7-23.6.56	6400
	57			F7-23.4.57	5400			F7-23.5.57	6400			F7-23.6.57	6400
	58			F7-23.4.58	5400			F7-23.5.58	6400			F7-23.6.58	6400
	59			F7-23.4.59	5400			F7-23.5.59	6400			F7-23.6.59	6400
	60			F7-23.4.60	5400			F7-23.5.60	6400			F7-23.6.60	3200
	61			F7-23.4.61	5400			F7-23.5.61	6400			F7-23.6.61	6400
	62			F7-23.4.62	5400			F7-23.5.62	6400			F7-23.6.62	6400
	63			F7-23.4.63	5400			F7-23.5.63	6400			F7-23.6.63	6400
	64			F7-23.4.64	5400			F7-23.5.64	6400			F7-23.6.64	
	65			F7-23.4.65	1800			F7-23.5.65	6400			F7-23.6.65	6400

Table 3: Piglets vaccinated at day 33 of age, the sows were inmunized only with MLV during the first third of pregnancy.

Identification		0 WPV Vaccination: First dose				3 WPV Vaccination: Second dose				7 WPV			
**Sows**	**Piglets**	**Date**	**Age**	**Sample Code**	**NAb Titers**	**Date**	**Age**	**Sample Code**	**NAb Titers**	**Date**	**Age**	**Sample Code**	**NAb Titers**
**125**	1021	02/02/2016	33 days, weanning	F7-41.1.1061	5	23/02/2016	56 days, weanning piglets	F7-41.2.1061	400	15/03/2016	77 days, weanning piglets	F7-41.3.1061	5400
	1066			F7-41.1.1066	5			F7-41.2.1066	200			F7-41.3.1066	5400
	1069			F7-41.1.1069	5			F7-41.2.1069	800			F7-41.3.1069	5400
	1070			F7-41.1.1070	5			F7-41.2.1070	200			F7-41.3.1070	5400
	1077			F7-41.1.1077	5			F7-41.2.1077	400			F7-41.3.1077	1800
	1079			F7-41.1.1079	5			F7-41.2.1079	200			F7-41.3.1079	5400
	1098			F7-41.1.1098	5			F7-41.2.1098	800			F7-41.3.1098	5400
**123**	1080	02/02/2016	33 days, weanning	F7-42.1.1080	5	23/02/2016	56 days, weanning piglets	F7-42.2.1080	400	15/03/2016	77 days, weanning piglets	F7-42.3.1080	5400
	1110			F7-42.1.1110	5			F7-42.2.1110	800			F7-42.3.1110	5400
	1113			F7-42.1.1113	25			F7-42.2.1113	400			F7-42.3.1113	5400
	1117			F7-42.1.1117	50			F7-42.2.1117	800			F7-42.3.1117	1800
	1120			F7-42.1.1120	5			F7-42.2.1120	800			F7-42.3.1120	5400
	1140			F7-42.1.1140	5			F7-42.2.1140	400			F7-42.3.1140	5400
	1150			F7-42.1.1150	5			F7-42.2.1150	200			F7-42.3.1150	5400
	1160			F7-42.1.1160	5			F7-42.2.1160	200			F7-42.3.1160	5400
**126**	1062	02/02/2016	33 days, weanning	F7-43.1.1062	5	23/02/2016	56 days, weanning piglets	F7-43.2.1062	400	15/03/2016	77 days, weanning piglets	F7-43.3.1062	5400
	1073			F7-43.1.1073	25			F7-43.2.1073	400			F7-43.3.1073	400
	1078			F7-43.1.1078	5			F7-43.2.1078	400			F7-43.3.1078	5400
	1081			F7-43.1.1081	25			F7-43.2.1081	800			F7-43.3.1081	5400
	1180			F7-43.1.1180	5			F7-43.2.1180	800			F7-43.3.1180	5400
